# Modeling and Analysis of a Conical Bridge-Type Displacement Amplification Mechanism Using the Non-Uniform Rational B-Spline Curve

**DOI:** 10.3390/ma16186162

**Published:** 2023-09-11

**Authors:** Mingze Wang, Cheng Zhang, Shutian Liu, Xiaoli Wang

**Affiliations:** State Key Laboratory of Structural Analysis, Optimization and CAE Software for Industrial Equipment, Dalian University of Technology, Dalian 116024, China; mzwang@mail.dlut.edu.cn (M.W.); andyzhang@mail.dlut.edu.cn (C.Z.); liess@mail.dlut.edu.cn (X.W.)

**Keywords:** piezoelectric actuator, compliant mechanisms, bridge-type displacement amplification mechanism, displacement amplification ratio, external load

## Abstract

This paper presents a new analytical model of a conical bridge-type displacement amplification mechanism (DAM) considering the effect of external loads and a piezostack actuator (PSA). With the merits of simple implementation and better fitting, the non-uniform rational B-spline (NURBS) is employed to parameterize conical connecting beams of the DAM, and an analytical model of the displacement amplification ratio and input stiffness is established based on Castigliano’s second theorem. After that, considering the interactions with elastic loads and PSA, the actual displacement amplification ratio of the conical DAM is obtained, and the effect of the shape of connecting beams in the performance of the DAM is further analyzed. The proposed analytical model is verified by finite element analysis (FEA), and the results show a maximum error of 6.31% between the calculated value and FEA results, demonstrating the accuracy of the proposed model. A prototype of the conical DAM with optimized shape is fabricated and experimentally tested, which further validates the effectiveness and accuracy of the proposed analytical model. The proposed model offers a new method for analysis and shape optimization of the bridge-type DAM under specific elastic loads.

## 1. Introduction

Due to the advantages of high rigidity, high bandwidth, and fast frequency response, piezostack actuators (PSAs) have been extensively used in micro-positioning [1,2,3], micro-manipulation [4,5,6], energy harvesting [7,8,9], aerospace engineering [10,11,12,13], optical alignment [14,15,16,17,18] and many other engineering fields [19,20]. Nevertheless, the main drawback of PSAs are their small stroke, which may not meet the requirements of most applications [21]. Therefore, various displacement amplification mechanisms (DAMs) have been developed to enlarge the output displacement of PSAs. Conventional DAMs can be divided into the following categories: lever-type [22], bridge-type [23,24], rhombus-type [25,26], and Scott–Russell-type DAMs [27]. Among the above mechanisms, the bridge-type and rhombus-type mechanisms have attracted significant interest due to their compact size and relatively large amplification ratio. Furthermore, numerous compound and multistage-compliant mechanisms with higher amplification ratios and modified natural frequencies have been developed based on these classic DAMs [28,29].

Since bridge-type and rhombus-type DAMs play essential roles in designing compliant mechanisms, it is necessary to establish an efficient and accurate analytical model for bridge-type and rhombus-type DAMs. Many analytical models have been developed to predict the displacement amplification ratio and stiffness of the bridge-type and rhombus-type DAMs. Lobontiu et al. [30] established an analytical model for the amplification ratio and stiffness of the bridge-type DAMs based on Castigliano’s second theorem, and the influence of the structural parameters on the amplification ratio and stiffness was further analyzed. Ma et al. [31] developed the analytical displacement amplification ratio using elastic beam theory. The accuracy of the theoretical model is relatively low, because the deformation of the connecting beam is not considered. Wei et al. [32] presented an analytical model of bridge-type mechanisms in general configurations utilizing the elastic beam theory, and based on the proposed model, a compliant bridge-type mechanism with higher displacement amplification ratio was optimized. Ling [33] derived a general static and dynamic analytical model of bridge-type amplifiers in three popular configurations. Moreover, a parameter influence analysis was performed and a comparison of the three bridge-type DAMs was conducted. Pan et al. [34] developed a nonlinear analytical model of both lumped and distributed bridge-type DAMs by regarding the connecting beam as a variable-thickness beam, and the influences of design parameters were evaluated. As the DAMs are usually connected to the PSA and specific elastic loads, many researchers have investigated the characteristic of the DAMs under external loads. Liu et al. [35] proposed an analytical model of displacement amplification ratio considering external loads, indicating that the displacement amplification ratio decreases significantly in the presence of external loads. Cao et al. [36] deduced the output displacement model of generic planar compliant mechanisms interacting with elastic loads and PSAs. Lin et al. [37] proposed an analytical model of the critical parameters of the precision positioning stage. The influence of critical parameters on geometric nonlinearity was concluded based on the proposed model. Recently, Tian et al. [38] presented a structure–load performance integrated-model method for bridge-type DAMs considering the deformations of all components, the effect of external load, and the nonlinear shear effect. The research indicated that the amplification ratio of bridge-type DAMs is strongly affected by external loads.

The studies mentioned above focused on modeling compliant amplification mechanisms with straight flexures and connecting beams. As a matter of fact, flexures and connecting beams can be of various shapes, such as elliptical [39] and of the Moonie type [40], which can significantly affect the performance of the amplification mechanism. Although the influence of some structural parameters, such as the thickness of flexures and the inclination angle of connecting beams, etc., on the DAMs have been evaluated in detail, the effect of the shape of the connecting beams on the performance of compliant mechanisms has not yet been thoroughly investigated. In addition, although various studies of the displacement amplification ratio of bridge-type DAMs under external loads have been presented, not as much attention has been paid to the relationship between the performance of DAMs and the stiffness of elastic external loads. In view of this, an analytical model of bridge-type DAMs with conical connecting beams under specific elastic loads is presented in this paper. The conical connecting beams are modeled based on non-uniform rational B-splines (NURBS), and an analytical model of the displacement amplification ratio and input stiffness is established based on Castigliano’s second theorem. Then, the theoretical model of the actual displacement amplification ratio of the conical DAM under interaction with elastic loads and a PSA is proposed. The accuracy of the analytical model is verified by employing finite element analysis (FEA), and the influence of the shape of the connecting beams on the displacement amplification ratio, stiffness and natural frequencies of the DAM is investigated. Subsequently, an optimal design for a conical bridge-type DAM under specific load stiffness is proposed, a prototype for which is further tested on the basis of experiments.

The rest of this paper is organized as follows: an analytical model of the conical DAM under interaction with elastic loads and PSA is deduced in Section 2. In Section 3, the proposed analytical model is verified via finite element analysis. Then, parameter analysis and shape optimization are carried out in Section 4, and a prototype of the optimal design is tested in Section 5. Finally, the conclusions of this paper are summarized in Section 6.

## 2. Analytical Modeling

### 2.1. Expression of Conical Curves Using NURBS

The NURBS curve, with the advantages of easy implementation in computation and better fitting performance, has been widely used in geometric modeling and computer-aided design (CAD) [41]. Many researchers have utilized the NURBS curve to optimize the topology and shape of compliant mechanisms [42,43]. The shape of the curve is determined by control points, the corresponding weights of the control points, and the order of the curve. In the Cartesian coordinate system, conic curves can be expressed using three control points:

(1) $$C\left(u\right)=\frac{{\left(1-u\right)}^{2}w_{0}P_{0}+2u\left(1-u\right)w_{1}P_{1}+u^{2}w_{2}P_{2}}{{\left(1-u\right)}^{2}w_{0}+2u\left(1-u\right)w_{1}+u^{2}w_{2}}$$

where $P_{0}=\left(x_{0},{\;y}_{0}\right)$, $P_{1}=\left(x_{1},{\;y}_{1}\right)$ and $P_{2}=\left(x_{2},{\;y}_{2}\right)$ represent the coordinates of three control points, $w_{0}$, $w_{1}$ and $w_{2}$ are the corresponding weights, and u is the parameterized variable in the range of $\left[0,1\right]$.

**Figure 1:**
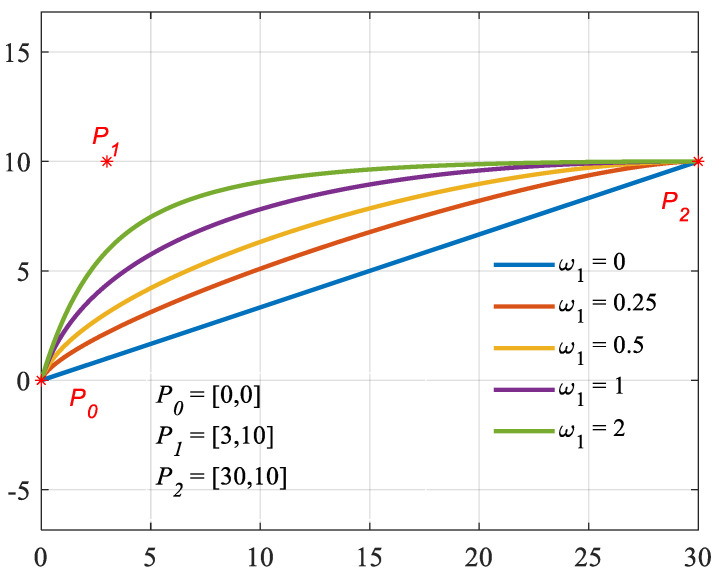
Conical curves based on NURBS.

If the first and third control points are fixed and let their weight is equal to one, the shape of the conical curves is determined by control point P_1_ and its weight $w_{1}$. As shown in Figure 1, when $w_{1}=0$, the curve is a straight line between P_0_ and P_2_. When $0<w_{1}<1$, the curve is elliptical. When $w_{1}=1$, the curve is parabolic. When $w_{1}>1$, the curve is hyperbolic.

### 2.2. Static Analysis of the DAM

**Figure 2:**
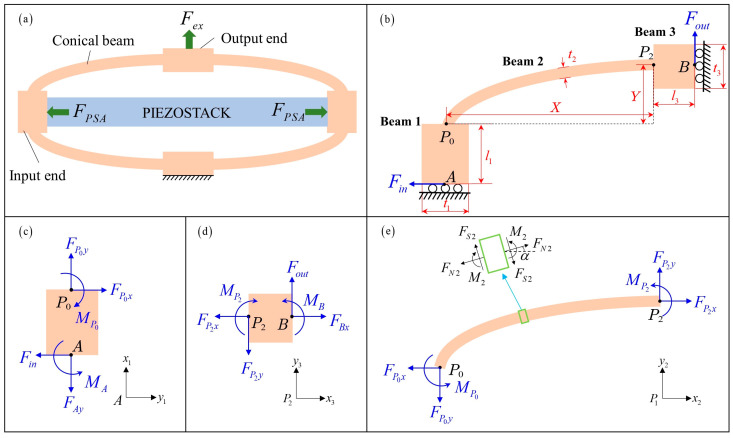
Analytical modeling of the conical-shape DAM. (**a**) Schematic diagram. (**b**) One-quarter model. (**c**) Input beam 1. (**d**) Output beam 3. (**e**) Conical connecting beam 2.

The schematic diagram of the conical DAM with PSA is shown in Figure 2. Only a quarter of the mechanism is needed to establish the analytical model because of the double symmetry. The input force $F_{\mathrm{in}}$ is half of the actuation force $F_{\mathrm{PSA}}$ generated by PSA and the output force $F_{\mathrm{out}}$ is half of the external force $F_{\mathrm{ex}}$. Considering the mechanical equilibrium, the reaction forces $F_{\mathrm{Ay}}$, $M_{A}$, $F_{\mathrm{Bx}}$, $M_{B}$ at endpoints A and B can be obtained as:

(2) $$\left\{ \begin{array}{l} F_{Ay}=F_{out}\\ F_{Bx}=F_{in}\\ M_{A}+M_{B}=F_{in}\cdot \left(Y+l_{1}\right)-F_{out}\cdot \left(X+l_{3}\right) \end{array}\right.$$

The force diagram and local coordinate system of beams 1–3 are shown in Figure 2c–e, respectively. The conical beam shown in Figure 2e can be expressed based on NURBS as:

(3) $$C\left(u\right)=\left[\begin{array}{l} x\left(u\right)\\ y\left(u\right) \end{array}\right]=\frac{1}{{\left(1-u\right)}^{2}+2u\left(1-u\right)w_{1}+u^{2}}\cdot \left[\begin{array}{l} {\left(1-u\right)}^{2}x_{0}+2u\left(1-u\right)w_{1}x_{1}+u^{2}x_{2}\\ {\left(1-u\right)}^{2}y_{0}+2u\left(1-u\right)w_{1}y_{1}+u^{2}y_{2} \end{array}\right]$$

The axial force $F_{\mathrm{Ni}}$, shear force $F_{Si}$ and bending moment $M_{i}$ of beam *i* (*i* = 1, 2, 3) can be calculated as:

(4) $$\left\{ \begin{array}{l} F_{N1}=F_{S3}=F_{out}\\ F_{N2}\left(u\right)=F_{in}\cdot \cos(\alpha )+F_{out}\cdot \sin(\alpha )\\ F_{S1}=F_{N3}=F_{in}\\ F_{S2}\left(u\right)=F_{in}\cdot \sin(\alpha )-F_{out}\cdot \cos(\alpha )\\ M_{1}\left(x_{1}\right)=F_{in}\cdot x_{1}-M_{A}\\ M_{2}\left(u\right)=F_{in}\cdot \left(y\left(u\right)+l_{1}\right)-F_{out}\cdot x\left(u\right)-M_{A}\\ M_{3}\left(x_{3}\right)=M_{B}+F_{out}\cdot \left(l_{3}-x_{3}\right) \end{array}\right.$$

where $\alpha =\arctan\;(y^{\prime }(u)/x^{\prime }(u))$ is the tilt angle of the conical beam at parameter *u*.

The strain energy of beam *i* = 1, 2, 3 can be obtained as follows:

(5) $$\left\{ \begin{array}{l} U_{1}=\int_{0}^{l_{1}}\left(\frac{F_{N1}^{2}}{2EA_{1}}+\frac{kF_{S1}^{2}}{2GA_{1}}+\frac{M_{1}^{2}}{2EI_{1}}\right)dx_{1}\\ U_{2}=\int_{C\left(u\right)}\left(\frac{F_{N2}^{2}}{2EA_{2}}+\frac{kF_{S2}^{2}}{2GA_{2}}+\frac{M_{2}^{2}}{2EI_{2}}\right)ds\\ U_{3}=\int_{0}^{l_{3}}\left(\frac{F_{N3}^{2}}{2EA_{3}}+\frac{kF_{S3}^{2}}{2GA_{3}}+\frac{M_{3}^{2}}{2EI_{3}}\right)dx_{3} \end{array}\right.$$

where *E* is Young’s modulus, and *G* is the shear modulus of the material. The parameter *k* represents the shape factor of the cross-section, the value of which for rectangular sections is 6/5. $A_{i}=bt_{i}$, $I_{i}=bt_{i}^{3}/12$ (*i* = 1, 2, 3) are the area and inertia moment of the cross-section of beam *i*. *b* and $t_{i}$ are the width of the DAM and the thickness of the beam *i*, respectively.

According to the geometric relations, the relationship between the infinitesimal segment of the conical beam and the parameter differential can be expressed as:

(6) $$ds=\sqrt{dx{\left(u\right)}^{2}+dy{\left(u\right)}^{2}}=D\left(u\right)\cdot du$$

where $D\left(u\right)=\sqrt{{x^{\prime }\left(u\right)}^{2}+{y^{\prime }\left(u\right)}^{2}}$.

The total strain energy of the DAM is the sum of the strain energies of the input beam, conical connecting beam, and output beam:

(7) $$U=U_{1}+U_{2}+U_{3}$$

Because of the symmetry condition of the mechanism, the rotation angle of point A and point B should remain zero. Therefore, the following equations can be derived from Castigliano’s second theorem:

(8) $$\left\{ \begin{array}{l} \theta_{A}=\frac{\partial U}{\partial M_{A}}=\int_{0}^{l_{1}}\frac{\partial M_{1}\left(x_{1}\right)}{\partial M_{A}}\cdot \frac{M_{1}\left(x_{1}\right)}{EI_{1}}dx_{1}+\int_{0}^{1}\frac{\partial M_{2}\left(u\right)}{\partial M_{A}}\cdot \frac{M_{2}\left(u\right)}{EI_{2}}D\left(u\right)du+\int_{0}^{l_{3}}\frac{\partial M_{3}\left(x_{3}\right)}{\partial M_{A}}\cdot \frac{M_{3}\left(x_{3}\right)}{EI_{3}}dx_{3}=0\\ \theta_{B}=\frac{\partial U}{\partial M_{B}}=\int_{0}^{l_{1}}\frac{\partial M_{1}\left(x_{1}\right)}{\partial M_{B}}\cdot \frac{M_{1}\left(x_{1}\right)}{EI_{1}}dx_{1}+\int_{0}^{1}\frac{\partial M_{2}\left(u\right)}{\partial M_{B}}\cdot \frac{M_{2}\left(u\right)}{EI_{2}}D\left(u\right)du+\int_{0}^{l_{3}}\frac{\partial M_{3}\left(x_{3}\right)}{\partial M_{B}}\cdot \frac{M_{3}\left(x_{3}\right)}{EI_{3}}dx_{3}=0 \end{array}\right.$$

Substituting Equations (4)–(7) into Equation (8), the relationship between the bending moment $M_{A}$, $M_{B}$ and the input, output force $F_{\mathrm{in}}$, $F_{\mathrm{out}}$ can be obtained as follows:

(9) $$\left[\begin{array}{c} M_{A}\\ M_{B} \end{array}\right]=\left[\begin{array}{cc} d_{11} & d_{12}\\ d_{21} & d_{22} \end{array}\right]\left[\begin{array}{c} F_{in}\\ F_{out} \end{array}\right]$$

where parameters $d_{11}$, $d_{12}$, $d_{21}$, $d_{22}$ can be calculated as:

$$\begin{array}{l} d_{11}=\frac{\left\{\frac{l_{1}^{2}}{2EI_{1}}+\frac{1}{EI_{2}}\left[\int_{0}^{1}y\left(u\right)D\left(u\right)du+l_{1}\int_{0}^{1}D\left(u\right)du\right]+\frac{l_{3}}{EI_{3}}\left(Y+l_{1}\right)\right\}}{\left(\frac{l_{1}}{EI_{1}}+\frac{1}{EI_{2}}\int_{0}^{1}D\left(u\right)du+\frac{l_{3}}{EI_{3}}\right)}\\ d_{12}=\frac{\left\{-\frac{1}{EI_{2}}\int_{0}^{1}x\left(u\right)D\left(u\right)du-\frac{l_{3}}{EI_{3}}\left(X+\frac{l_{3}}{2}\right)\right\}}{\left(\frac{l_{1}}{EI_{1}}+\frac{1}{EI_{2}}\int_{0}^{1}D\left(u\right)du+\frac{l_{3}}{EI_{3}}\right)}\\ d_{21}=Y+l_{1}-d_{11}\\ d_{22}=-\left(X+l_{3}\right)-d_{12} \end{array}$$

The displacement of the amplification mechanism at the input end and output end can be obtained by Castigliano’s second theorem:

(10) $$\begin{array}{l} \Delta x=\frac{\partial U}{\partial F_{in}}=\int_{0}^{l_{1}}\frac{\partial F_{N1}}{\partial F_{in}}\cdot \frac{F_{N1}}{EA_{1}}dx_{1}+\int_{0}^{l_{1}}\frac{\partial F_{S1}}{\partial F_{in}}\cdot \frac{kF_{S1}}{GA_{1}}dx_{1}+\int_{0}^{l_{1}}\frac{\partial M_{1}\left(x_{1}\right)}{\partial F_{in}}\cdot \frac{M_{1}\left(x_{1}\right)}{EI_{1}}dx_{1}\\ +\int_{0}^{1}\frac{\partial F_{N2}}{\partial F_{in}}\cdot \frac{F_{N2}}{EA_{2}}\cdot D\left(u\right)du+\int_{0}^{1}\frac{\partial F_{S2}}{\partial F_{in}}\cdot \frac{F_{S2}}{EA_{2}}\cdot D\left(u\right)du+\int_{0}^{1}\frac{\partial M_{2}\left(u\right)}{\partial F_{in}}\cdot \frac{M_{2}\left(u\right)}{EI_{2}}\cdot D\left(u\right)du\\ +\int_{0}^{l_{3}}\frac{\partial F_{N3}}{\partial F_{in}}\cdot \frac{F_{N3}}{EA_{3}}dx_{3}+\int_{0}^{l_{3}}\frac{\partial F_{S3}}{\partial F_{in}}\cdot \frac{kF_{S3}}{GA_{3}}dx_{3}+\int_{0}^{l_{3}}\frac{\partial M_{3}\left(x_{3}\right)}{\partial F_{in}}\cdot \frac{M_{3}\left(x_{3}\right)}{EI_{3}}dx_{3} \end{array}$$

(11) $$\begin{array}{l} \Delta y=\frac{\partial U}{\partial F_{out}}=\int_{0}^{l_{1}}\frac{\partial F_{N1}}{\partial F_{out}}\cdot \frac{F_{N1}}{EA_{1}}dx_{1}+\int_{0}^{l_{1}}\frac{\partial F_{S1}}{\partial F_{out}}\cdot \frac{kF_{S1}}{GA_{1}}dx_{1}+\int_{0}^{l_{1}}\frac{\partial M_{1}\left(x_{1}\right)}{\partial F_{out}}\cdot \frac{M_{1}\left(x_{1}\right)}{EI_{1}}dx_{1}\\ +\int_{0}^{1}\frac{\partial F_{N2}}{\partial F_{out}}\cdot \frac{F_{N2}}{EA_{2}}\cdot D\left(u\right)du+\int_{0}^{1}\frac{\partial F_{S2}}{\partial F_{out}}\cdot \frac{F_{S2}}{EA_{2}}\cdot D\left(u\right)du+\int_{0}^{1}\frac{\partial M_{2}\left(u\right)}{\partial F_{out}}\cdot \frac{M_{2}\left(u\right)}{EI_{2}}\cdot D\left(u\right)du\\ +\int_{0}^{l_{3}}\frac{\partial F_{N3}}{\partial F_{out}}\cdot \frac{F_{N3}}{EA_{3}}dx_{3}+\int_{0}^{l_{3}}\frac{\partial F_{S3}}{\partial F_{out}}\cdot \frac{kF_{S3}}{GA_{3}}dx_{3}+\int_{0}^{l_{3}}\frac{\partial M_{3}\left(x_{3}\right)}{\partial F_{out}}\cdot \frac{M_{3}\left(x_{3}\right)}{EI_{3}}dx_{3} \end{array}$$

Simplifying Equations (10) and (11), the input displacement ∆x and output displacement ∆y can be further expressed in matrix form:

(12) $$\left[\begin{array}{l} \Delta x\\ \Delta y \end{array}\right]=\left[\begin{array}{cc} c_{11} & c_{12}\\ c_{21} & c_{22} \end{array}\right]\left[\begin{array}{c} F_{in}\\ F_{out} \end{array}\right]$$

The parameters $c_{11}$, $c_{12}$, $c_{21}$, $c_{22}$ in Equation (12) can be calculated as follows:

(13) $$\begin{array}{l} c_{11}=\frac{1}{K_{s1}}+\frac{1}{K_{\theta 1}}\left(\frac{l_{1}^{2}}{3}-l_{1}d_{11}+d_{11}^{2}\right)+\frac{1}{K_{l3}}+\frac{1}{K_{\theta 3}}d_{21}^{2}+\frac{1}{{K^{\prime }}_{l2}}\frac{a_{2}}{{l^{\prime }}_{2}}+\frac{1}{{K^{\prime }}_{s2}}\frac{a_{1}}{{l^{\prime }}_{2}}+\frac{1}{{K^{\prime }}_{\theta 2}}\frac{a_{4}}{{l^{\prime }}_{2}}\\ c_{12}=c_{21}=-\frac{1}{K_{\theta 1}}\left(\frac{l_{1}}{2}-d_{11}\right)d_{12}+\frac{1}{K_{\theta 3}}\left(d_{22}+\frac{l_{3}}{2}\right)d_{21}+\frac{1}{{K^{\prime }}_{l2}}\frac{a_{3}}{{l^{\prime }}_{2}}-\frac{1}{{K^{\prime }}_{s2}}\frac{a_{3}}{{l^{\prime }}_{2}}-\frac{1}{{K^{\prime }}_{\theta 2}}\frac{a_{6}}{{l^{\prime }}_{2}}\\ c_{22}=\frac{1}{K_{l1}}+\frac{1}{K_{\theta 1}}d_{12}^{2}+\frac{1}{K_{s3}}+\frac{1}{K_{\theta 3}}\left(\frac{l_{3}^{2}}{3}+l_{3}d_{22}+d_{22}^{2}\right)+\frac{1}{{K^{\prime }}_{l2}}\frac{a_{1}}{{l^{\prime }}_{2}}+\frac{1}{{K^{\prime }}_{s2}}\frac{a_{2}}{{l^{\prime }}_{2}}+\frac{1}{{K^{\prime }}_{\theta 2}}\frac{a_{5}}{{l^{\prime }}_{2}} \end{array}$$

where $K_{\mathrm{li}}=EA_{i}/l_{i}$, $K_{\mathrm{si}}=GA_{i}/kl_{i}$ and $K_{\theta i}=EI_{i}/l_{i}$ $\left(i=1,3\right)$ are the axial tensile stiffness, shear stiffness and rotational stiffness of the input and output beam, respectively. $l_{2}^{\prime }=\sqrt{X^{2}+Y^{2}}$ is the equivalent length of the conical beam, $K_{l2}^{\prime }=EA_{2}\;/{\;l}_{2}^{\prime }$, $K_{s2}^{\prime }=GA_{2}\;/{kl}_{2}^{\prime }$ and $K_{\theta 2}^{\prime }=EI_{2}\;/{\;l}_{2}^{\prime }$ are the equivalent axial tensile stiffness, shear stiffness and rotational stiffness of the conical beam, respectively.

The integrals $a_{1}$, $a_{2}$, $a_{3}$, $a_{4}$, $a_{5}$ and $a_{6}$ in Equation (13) can be calculated using the numerical integration function ‘integral’ in MATLAB:

(14) $$\left\{ \begin{array}{l} a_{1}=\int_{0}^{1}\sin^{2}\alpha \cdot D\left(u\right)du,\;a_{2}=\int_{0}^{1}\cos^{2}\alpha \cdot D\left(u\right)du,\;a_{3}=\int_{0}^{1}\sin\alpha \cdot \cos\alpha \cdot D\left(u\right)du\\ a_{4}=\int_{0}^{1}{\left(y\left(u\right)+l_{1}-d_{11}\right)}^{2}\cdot D\left(u\right)du,\;a_{5}=\int_{0}^{1}{\left(x\left(u\right)+d_{12}\right)}^{2}\cdot D\left(u\right)du\\ a_{6}=\int_{0}^{1}\left(y\left(u\right)+l_{1}-d_{11}\right)\cdot \left(x\left(u\right)+d_{12}\right)\cdot D\left(u\right)du \end{array}\right.$$

The displacement amplification ratio of the conical shape amplification mechanism can be obtained as follows:

(15) $$R=\left|\frac{\delta_{out}}{\delta_{in}}\right|=\frac{\left|c_{21}F_{in}+c_{22}F_{out}\right|}{\left|c_{11}F_{in}+c_{12}F_{out}\right|}$$

where $\delta_{\mathrm{in}}=2∆x$, $\delta_{\mathrm{out}}=2∆y$ are the total input and output displacement of the amplification mechanism, respectively.

When no external load is acting on the output end of the amplification mechanism, the inherent displacement amplification ratio and input stiffness of the amplification mechanism can be calculated using Equation (16), the value of which is only related to the size and material parameters of the mechanism.

(16) $$R_{0}=\left|\frac{c_{21}}{c_{11}}\right|,\;K_{in}=\frac{1}{c_{11}}$$

### 2.3. Analytical Model Considering the Effect of PSA and External Load

**Figure 3:**
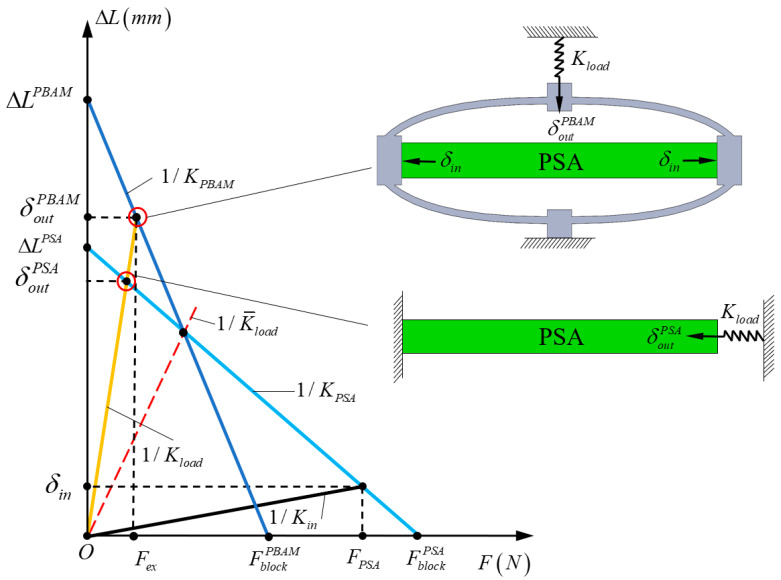
The working principle of the DAM with PSA and elastic load.

The DAM is installed outside the PSA, and in most applications, elastic loads such as guiding mechanisms are connected to the output end of the mechanism. The actual output displacement is determined not only by the characteristics of the DAM, but also by the properties of PSA and the stiffness of the elastic load. Therefore, it is significant to incorporate the effect of elastic loads and PSA into analytical models. The working principle of the DAM with PSA and elastic load is shown in Figure 3. The output displacement of PSA at no external load can be obtained as:

(17) $$\Delta L^{PSA}=K_{V}V$$

where $K_{V}=\;n\cdot d_{33}$ is the piezoelectric constant, n represents the number of piezoceramic sheets, $d_{33}$ is the piezoelectric coefficient, and V is the driving voltage of the PSA.

When the PSA is connected to the DAM, the output displacement of the PSA, which is also the input displacement of the DAM, can be expressed as:

(18) $$\delta_{in}=\frac{K_{PSA}}{K_{PSA}+K_{in}}\Delta L^{PSA}$$

where $K_{\mathrm{PSA}}$ is the axial stiffness of the PSA, and $K_{\mathrm{in}}$ is the input stiffness of the DAM.

The actuation force generated by PSA $F_{\mathrm{PSA}}$ can be obtained as follows:

(19) $$F_{PSA}=K_{PSA}\cdot \left(\Delta L^{PSA}-\delta_{in}\right)$$

As can be seen in Figure 3, the piezo-based actuation mechanism (PBAM) composed of the PSA and the DAM can be regarded as a PSA actuator with amplified free displacement $∆L^{\mathrm{PBAM}}=R_{0}\cdot \delta_{\mathrm{in}}$ and attenuated block force $F_{\mathrm{block}}^{\mathrm{PBAM}}$. When elastic load with stiffness $K_{\mathrm{load}}$ is applied to the output end of the PBAM, the output displacement of the PBAM can be expressed as:

(20) $$\delta_{out}^{PBAM}=\frac{K_{PBAM}}{K_{PBAM}+K_{load}}\cdot \Delta L^{PBAM}=\frac{K_{PBAM}}{K_{PBAM}+K_{load}}\cdot R_{0}\cdot \frac{K_{PSA}}{K_{in}+K_{PSA}}\cdot K_{V}\cdot V$$

where $K_{\mathrm{PBAM}}=F_{\mathrm{block}}^{\mathrm{PBAM}}/∆L^{\mathrm{PBAM}}$ is the equivalent stiffness of the PBAM.

Substituting Equation (19) into Equation (12) and setting $\delta_{\mathrm{out}}=0$ in Equation (12), the block force of the PBAM can be calculated as follows:

(21) $$F_{block}^{PBAM}=\frac{c_{21}\cdot K_{PSA}}{c_{22}+\left(c_{11}c_{22}-c_{12}c_{21}\right)K_{PSA}}\cdot \Delta L^{PSA}$$

Then, the equivalent stiffness of the PBAM $K_{\mathrm{PBAM}}$ can be derived as:

(22) $$K_{PBAM}=\frac{F_{block}^{PBAM}}{\Delta L^{PBAM}}=\frac{1+c_{11}K_{PSA}}{c_{22}+\left(c_{11}c_{22}-c_{12}c_{21}\right)K_{PSA}}$$

When an elastic load of the same stiffness is applied directly to the PSA, the output displacement of the PSA can be obtained as:

(23) $$\delta_{out}^{PSA}=\frac{K_{PSA}}{K_{PSA}+K_{load}}\Delta L^{PSA}$$

To better evaluate the effectiveness of the DAM under the elastic load, the actual displacement amplification ratio $R_{\mathrm{act}}$ is defined as the ratio of the output displacement of the PBAM to the output displacement of the PSA under the same elastic load:

(24) $$R_{act}=\frac{\delta_{out}^{PBAM}}{\delta_{out}^{PSA}}=\frac{K_{PBAM}}{K_{PBAM}+K_{load}}\cdot \frac{K_{PSA}+K_{load}}{K_{PSA}+K_{in}}\cdot R_{0}$$

According to the principle of conservation of energy, there is always an intersection between the force–displacement curves of the PBAM and PSA. As shown in Figure 3, the red dashed line is obtained by connecting the intersection point to the origin of coordinates, the slope of which is the inverse of the critical load stiffness ${\bar{K}}_{\mathrm{load}}$. When the load stiffness is below the critical load stiffness and the output displacement of the PBAM is larger than the output displacement of the PSA, that is, $R_{\mathrm{act}}>1$, the DAM is considered efficient under elastic load. When the load stiffness is equal to the critical stiffness and the output displacement of the PBAM is equal to that of the PSA, that is, $R_{\mathrm{act}}=1$, the DAM only changes the direction of the motion. When the load stiffness is greater than the critical load stiffness and the output displacement of the PBAM is smaller than the output displacement of the PSA, that is, $R_{\mathrm{act}}<\;1$, the displacement amplification mechanism is considered inefficient under the elastic load.

## 3. FEA Verification

In this section, the established analytical models are verified by means of finite element analysis. To make the verification sufficient, eight samples with different materials, control points, weights, and load stiffness are constructed. The control point $P_{0}$ of the six samples is fixed at $\left(0,\;0\right)$, and the control points of the connecting beam 2 $P_{1}$, $P_{2}$, weight $w_{1}$, and stiffness of the external loads are tabulated in Table 1. The length, width, thickness of beams 1 and 3, and the thickness of beam 2 are set to be the same, as shown in Table 2. The physical and mechanical properties of the alloy Al-6061 and the structural steel are listed in Table 3. The 3D models of the samples are created using SOLIDWORKS, and the finite element analysis is carried out by employing the commercial software ANSYS Workbench2020R2. Adaptive mesh division is adopted for accurate modeling. 

**Figure 4:**
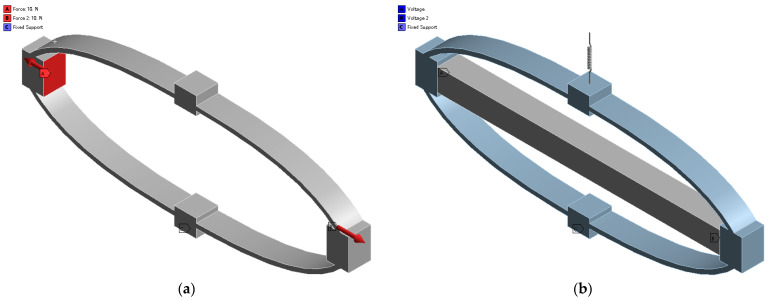
Finite element models: (**a**) the DAM; (**b**) the PBAM with the elastic load.

As shown in Figure 4, the DAM, the PBAM, and the PSA with elastic load are modeled separately to evaluate the inherent properties of the DAM ($R_{0}$, $K_{\mathrm{in}}$), the stiffness of the PBAM $K_{\mathrm{PBAM}}$, and the actual displacement amplification ratio $R_{\mathrm{act}}$, respectively. When simulating the DAM, the bottom of the DAM is fixed, and two concentrated forces of 10 N are applied to the input ends. As for the simulation of the PBAM, the PSA is modeled using the ‘Piezo And MEMS’ extension. The length, width, and thickness of the PSA are $l_{\mathrm{PSA}}=128\;\mathrm{mm}$, $b_{\mathrm{PSA}}=10\;\mathrm{mm}$, $t_{\mathrm{PSA}}=10\;\mathrm{mm}$, respectively. The material type is PZT-5H and the properties of the PSA are given as n=1190, $d_{33}=8.1\;\times \;10^{-10}\;C/N$, $K_{\mathrm{PSA}}=38\;N/µm$. The equivalent excitation voltage of 120 V × 1190 is imposed on two sides of the PSA. The bottom of the PBAM is fixed, and the free stroke of the output end is obtained first; then, the blocking force is achieved by fixing the output end of the PBAM. The equivalent stiffness of the PBAM can be obtained through the block force divided by the free stroke.

The inherent displacement amplification ratio $R_{0}$, input stiffness $K_{\mathrm{in}}$, stiffness of the PBAM $K_{\mathrm{PBAM}}$, and the actual displacement amplification ratio $R_{\mathrm{act}}$ are calculated by the analytical model and compared with the FEA results, as shown in Table 4. It can be seen that the deviations between the analytical and FEA results are smaller than 6.31%, indicating that the analytical model has high calculation accuracy and can guide the optimal design of the DAM under specific elastic loads.

It should be noted that the material of the samples has little influence on the inherent displacement amplification ratio $R_{0}$ of the DAMs, while it has a relatively large influence on the input stiffness $K_{\mathrm{in}}$, equivalent stiffness $K_{\mathrm{PBAM}}$, and actual displacement amplification ratio $R_{\mathrm{act}}$. The stiffer the material used, the greater the input stiffness $K_{\mathrm{in}}$, equivalent stiffness $K_{\mathrm{PBAM}}$, and actual displacement amplification ratio $R_{\mathrm{act}}$.

## 4. Parameter Analysis and Optimization

In this section, the influence of the shape of connecting beam 2 on the actual displacement amplification ratio is first analyzed utilizing the formulated analytical model. Then, an optimum architectural design of the DAM is determined under specific load stiffness. Considering the size of the PSA and manufacturing restrictions, the control points P_0_ and P_2_ are fixed at (0, 0), (64, 16), respectively. Control point $P_{1}=\left(x_{1},{\;y}_{1}\right)$ and its weight $w_{1}$ are variable parameters, the upper and lower bounds of which are given as: $0\leq x_{1}\leq 32$, $0\leq {\;y}_{1}\;\leq 16$, $0\leq w_{1}\leq 2$. The other geometric parameters are listed in Table 2. The material of the DAM is Al-6061, and its properties are shown in Table 3.

**Figure 5:**
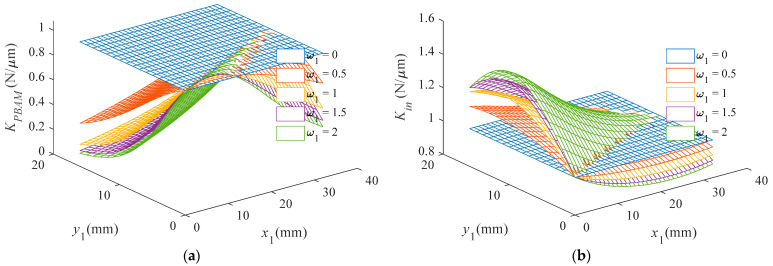
The changes in critical properties with the shape of connecting beams varied. (**a**) Equivalent stiffness of the PBAM. (**b**) Input stiffness.

The variation in the equivalent stiffness of the PBAM $K_{\mathrm{PBAM}}$ and input stiffness $K_{\mathrm{in}}$ with the variable parameters $\left(x_{1},{\;y}_{1},w_{1}\right)$ is shown in Figure 5. It is easy to observe that the equivalent stiffness of the PBAM decreases sharply when the weight $w_{1}$ increases from 0 to 2, and the maximum equivalent stiffness can be reached when the connecting beam is nearly straight. The input stiffness increases with increasing $w_{1}$ when the connecting beam is convex (${\;y}_{1}>\;x_{1}/4$) and decreases with increasing $w_{1}$ when the connecting beam is concave (${\;y}_{1}\;<\;x_{1}/4$). Moreover, the input stiffness decreases when $x_{1}$ increases. As ${\;y}_{1}$ increases, the input stiffness first increases and then decreases.

**Figure 6:**
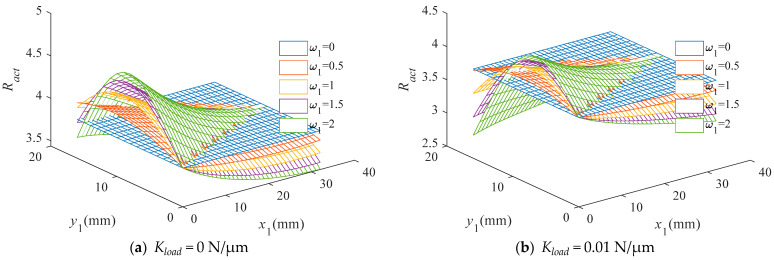

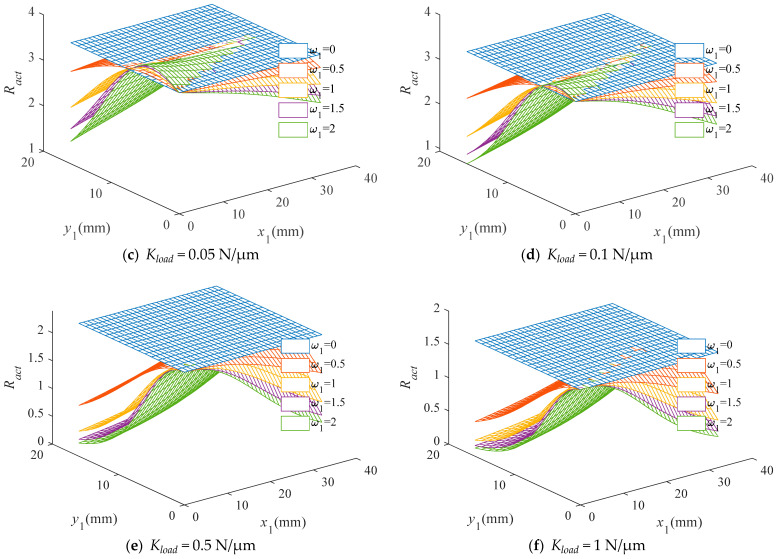
The actual amplification ratio in the case of different elastic loads.

The relationships between the actual displacement amplification ratio and the variable parameters $\left(x_{1},{\;y}_{1},w_{1}\right)$ under different elastic loads are shown in Figure 6. It can be seen that the actual displacement amplification ratio is significantly influenced by the shape of the connecting beams. Moreover, the optimal shape of the connecting beam is different under different load stiffness: for low-load-stiffness conditions ($K_{\mathrm{load}}\leq 0.1\;N/µm$), the actual displacement amplification ratio reaches its maximum when $w_{1}=2$, which means that connecting beams with hyperbolic shapes can make the DAM more efficient. Additionally, as $K_{\mathrm{load}}$ increases, the optimal control point $P_{1}$ moves toward the origin point. In the case of high load stiffness ($K_{\mathrm{load}}\;>0.1\;N/µm$), as $w_{1}$ increases, the actual displacement amplification ratio decreases, and the conventional rhombus-type DAM with straight connecting beams has better performance.

Having verified the influence of the shape of the connecting beam on the displacement amplification ratio, the bridge-type DAM connected to elastic loads with $K_{\mathrm{load}}=0.01\;N/µm$ is optimized in order to maximize the displacement amplification ratio. The optimization is implemented using the global optimization toolbox in MATLAB, and the optimized parameters are: $P_{1}=\left(0\;\mathrm{mm},\;6.8\;\mathrm{mm}\right)$, and $w_{1}=2$. The optimal actual displacement amplification ratio is 4.47.

## 5. Experimental Testing

**Figure 7:**
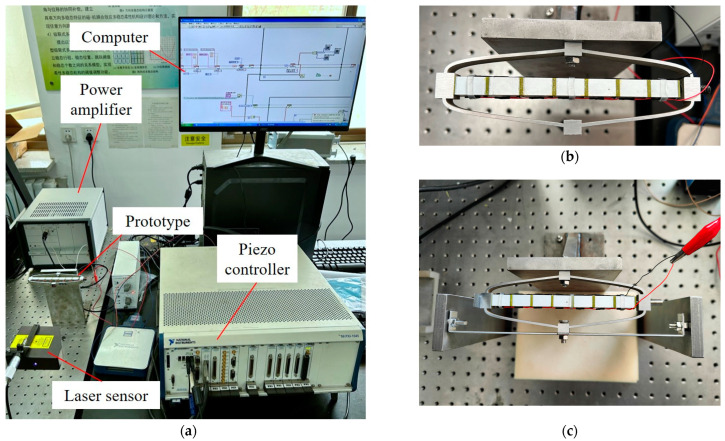
(**a**) Experimental setup. (**b**) Prototype without elastic load. (**c**) Prototype with 0.01 N/µm elastic load.

To further verify the feasibility of the proposed model, a prototype with optimal parameters is fabricated and tested under elastic load. The experimental setup and a close view of the prototype are illustrated in Figure 7a. The DAM is manufactured by means of the wire electrical discharge process using a piece of Al-6061 alloy. A PSA (PSt150/10 × 10/140, COREMORROW, Inc., Harbin, China) is inserted into the DAM and preloaded by a bolt between the PSA and the input end of the mechanism. The specifications of the PSA are listed in Table 5. Driving signals generated by the piezo controller (NI PXI-8110, National Instruments Corp., Austin, TX, USA) are transmitted to the power amplifier (LA75, CEDRAT TECHNOLOGIES, Meylan, France) with an output voltage of 0~150 V and an amplification ratio of 10. The output displacement is measured by a laser displacement sensor (LTS-200/100, MTI Instruments Incorporated, Albany, NY, USA) with a 100 mm measurement range and 1.9 µm resolution.

**Figure 8:**
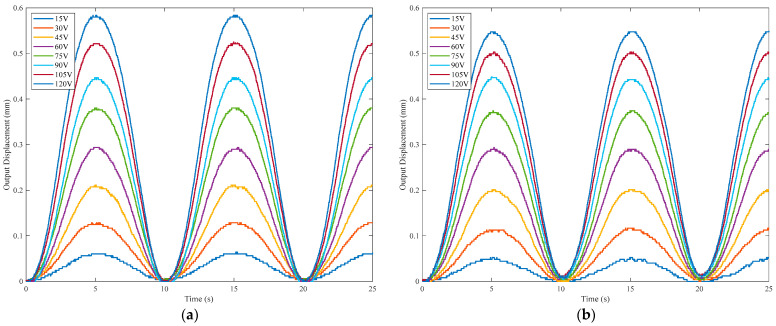
Experimental results of output displacement: (**a**) no load; (**b**) 0.01 N/µm elastic load.

**Figure 9:**
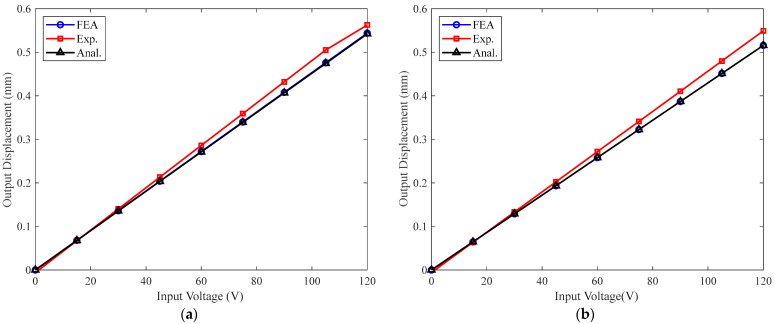
Comparisons of output displacement obtained using three methods: (**a**) no load; (**b**) 0.01 N/µm elastic load.

The prototype is tested under no load and 0.01 N/µm elastic load, respectively, as illustrated in Figure 7b,c. For the test of the amplification ratio under elastic load, the output end of the prototype is rigidly connected to an elastic sheet with equivalent stiffness of 0.01 N/µm. Both ends of the elastic sheet are fixed on the rigid frame. A 0.1 Hz sinusoidal voltage with a peak-to-peak amplitude of 0~120 V is applied to the PSA, and the output displacements of the prototype under two test cases (no load, 0.01 N/µm elastic load) are shown in Figure 8. It can be seen from the results that the output displacement of the PBAM under elastic load is slightly lower than the displacement with the free output end. The output displacements obtained by analytical, FEA and experimental methods are plotted in Figure 9 for comparison purposes. It can be observed that the analytical results deviate less than 0.3% and 3.6% from the FEA and experimental results, respectively, which shows the high accuracy of the proposed analytical model.

## 6. Conclusions

In this paper, a new analytical model of the conical bridge-type DAM was developed. To efficiently model the bridge-type DAM with conical connecting beams, the NURBS curve was employed to parameterize the conical connecting beam of the DAM. Considering the interactions between the external loads, PSA and DAM, static analytical models of the displacement amplification ratio and input stiffness were established based on Castigliano’s second theorem. Based on the proposed model, the relationship between the performance of the DAM and the shape of the connecting beams was investigated in depth. FEA was performed to validate the analytical model, and the maximum deviation between analytical and FEA results was 6.31%. To further verify the analytical model, a prototype of the conical DAM with optimized shape was fabricated and tested. The analytical results matched well with the FEA and experimental results, and the maximum errors were less than 0.3% and 3.6%, respectively. The proposed model is sufficiently efficient and accurate, and can be used to predict and further optimize bridge-type DAMs under different loading conditions.

## Figures and Tables

**Table 1:** Materials and dimensional parameters of eight samples.

Samples	Materials	$\mathrm{Weights}\;w_{1}$	Control Points $P_{1}$ (mm)	Control Points $P_{2}$ (mm)	Load Stiffness $K_{\mathrm{load}}$ (N/µm)
1	Structural steel	0.5	(30,16)	(64,16)	0.05
2	Al-6061	0.5	(16,16)	(64,16)	0.01
3	Structural steel	1	(16,16)	(64,16)	0.01
4	Al-6061	1	(5,16)	(64,16)	0.01
5	Al-6061	1	(16,10)	(64,10)	0.05
6	Structural steel	1.5	(16,16)	(64,16)	0.05
7	Structural steel	2	(16,16)	(64,16)	0.01
8	Al-6061	2	(16,16)	(64,16)	0.01

**Table 2:** Geometric parameters of beams 1–3.

Parameters	$l_{1}\left(\mathrm{mm}\right)$	$l_{3}\left(\mathrm{mm}\right)$	$t_{1}\left(\mathrm{mm}\right)$	$t_{2}\left(\mathrm{mm}\right)$	$t_{3}\left(\mathrm{mm}\right)$	b (mm)
Values	7	5	10	2	8	10

**Table 3:** Physical and mechanical properties of the alloy Al-6061 and structural steel.

Materials	Young’s Modulus *E* (GPa)	Poisson′s Ratio µ	Density ρ $(\mathrm{kg}/m^{3})$
Al-6061	69	0.33	2713
Structural steel	200	0.3	7850

**Table 4:** Comparisons between analytical and FEA results of different samples.

Samples	$R_{0}$			$K_{\mathrm{in}}$ (N/µm)			$K_{\mathrm{PBAM}}$ (N/µm)			$R_{\mathrm{act}}$		
	FEA	Anal.	Error (%)	FEA	Anal.	Error (%)	FEA	Anal.	Error (%)	FEA	Anal.	Error (%)
1	3.87	3.82	1.38	0.94	0.89	5.32	0.84	0.84	0.48	3.58	3.53	1.42
2	3.95	3.92	0.67	0.33	0.33	1.38	0.22	0.23	2.10	3.76	3.72	0.67
3	3.82	3.80	0.60	0.97	0.96	0.75	0.29	0.30	2.06	3.62	3.59	0.80
4	3.94	3.99	1.32	0.36	0.38	4.42	0.07	0.07	3.71	3.43	3.48	1.51
5	6.13	5.98	2.59	0.95	0.89	6.31	0.09	0.09	0.13	3.93	3.83	2.72
6	3.69	3.66	0.10	0.97	0.95	1.13	0.21	0.22	1.93	2.91	2.92	0.68
7	3.57	3.54	0.97	1.00	0.95	5.12	0.18	0.18	1.89	3.30	3.28	0.63
8	3.58	3.54	0.99	0.33	0.33	1.68	0.06	0.06	0.14	3.08	3.04	1.35

**Table 5:** Specifications of the PSA.

Parameters	Values	Parameters	Values
Dimensions (mm)	10 × 10 × 128	Stiffness (N/µm)	38
Maximum displacement (µm)	143.1	Electrical capacitance (µF)	50.4

## Data Availability

The data presented in this study are available on request from the corresponding author. The data are not publicly available due to the data also forming part of an ongoing study.
